# Potentiation effect of mallotojaponin B on chloramphenicol and mode of action of combinations against Methicillin-resistant *Staphylococcus aureus*

**DOI:** 10.1371/journal.pone.0282008

**Published:** 2023-03-21

**Authors:** Branly-Natalien Nguena-Dongue, Joseph Tchamgoue, Yvan Anderson Ngandjui Tchangoue, Paul Keilah Lunga, Kouipou Rufin Marie Toghueo, Menkem Elisabeth Zeuko`o, Yanick Kevin Dongmo Melogmo, Jean Claude Tchouankeu, Simeon Fogue Kouam, Boyom Fabrice Fekam

**Affiliations:** 1 Antimicrobial and Biocontrol Agents Unit (AmBcAU), Laboratory for Phytobiochemistry and Medicinal Plants Studies, Department of Biochemistry, Faculty of Science, University of Yaoundé I, Yaoundé, Cameroon; 2 Department of Organic Chemistry, Faculty of Science, University of Yaoundé I, Yaoundé, Cameroon; 3 Department of Chemistry, Higher Teacher Training College, University of Yaounde I, Yaounde, Cameroon; Universidad Autonoma de Chihuahua, MEXICO

## Abstract

*Staphylococcus aureus*, the causative agent of many infectious diseases has developed resistance to many antibiotics, even chloramphenicol which was the essential antibiotic recommended for the treatment of bacterial infection. Thus, other alternatives to fight against *S*. *aureus* infections are necessary; and combinatory therapy of antibiotics with natural compounds is one of the approaches. This study evaluated the activity of the combination of mallotojaponin B and chloramphenicol against Methicillin-resistant *Staphylococcus aureus* (MRSA). Antibacterial activities were evaluated by broth microdilution and the checkerboard methods. Modes of action as time-kill kinetic, Nucleotide leakage, inhibition and eradication of biofilm, and loss of salt tolerance were evaluated. Cytotoxicity was evaluated on Vero and Raw cell lines. Mallotojaponin B showed good activity against MRSA with a MIC value of 12.5 μg/mL. MRSA showed high resistance to chloramphenicol (MIC = 250 μg/mL). The combination produced a synergistic effect with a mean FICI of 0.393. This combination was bactericidal, inducing nucleotide leakage, inhibiting biofilm formation, and eradicating biofilm formed by MRSA. The synergic combination was non-cytotoxic to Vero and Raw cell lines. Thus, the combination of mallotojaponin B and chloramphenicol could be a potential alternative to design a new drug against MRSA infections.

## Introduction

*Staphylococcus aureus* is a commensal bacterium and a major human pathogen responsible for 30% of infections. It is the causative agent of skin and soft tissue infections, eye infections, sepsis, osteomyelitis, endocarditis, pneumonia, gastroenteritis, meningitis, toxic shock syndrome, and urinary tract infections [1]. *S*. *aureus* is known to be resistant to some antibiotics. Methicillin-resistant *Staphylococcus aureus* (MRSA) is classified among the 12 deadliest and most drug-resistant bacteria by the WHO [2]. It was responsible for more than 19 thousand and 157 thousand deaths in the USA and Europe respectively [3]. In Africa, the prevalence is heterogenetic and depends on each country [4]. In Congo and Ethiopia for example, the prevalence of MRSA is 63.5% and 37.43% respectively [4]. In Cameroon, the data are limited, but a prevalence of 72% and 80% was noted at the Military Hospital and the Central Hospital of Yaoundé respectively [5, 6].

Chemotherapy is the strategy for the management of MRSA infections which consists of the use of recommended antibiotics [7]. Nevertheless, this strategy has several limitations as side effects such as hepatotoxicity, nephrotoxicity, carcinogenic effects, and bone marrow aplasia often develop. In addition, MRSA strains are becoming increasingly resistant to some classes of antibiotics [8], and this represents one of the public health challenges. Therefore, there is an urgent need to search for other avenues for the treatment of *S*. *aureus* infection.

Natural products play an important role in the development of drugs against human diseases. Indeed, about 119 chemical substances from medicinal plants are used in several countries nowadays. Amongst these drugs, 74% were isolated from medicinal plants during phytochemical studies [9]. Thus, in our previous studies some phloroglucinols, including mallotojaponin B, were isolated from *Mallotus oppositifolius* and shown to possess interesting antibacterial activities [10]. Since combinatory therapy is one of the approaches in designing a new drug, the combination of natural compounds between them or with available antibiotics would be an appropriate means to fight against multi-resistant bacteria [11, 12]. In this way, this study aimed to evaluate the activity of the combination of phloroglucinol with an antibiotic and explore some modes of action of the best combinations against methicillin-resistant *Staphylococcus aureus* ATCC 33591. The phloroglucinol (mallotojaponin B) was chosen from amongst other phloroglucinols based on its potent activity against the MRSA, while chloramphenicol was selected from other antibiotics because great resistance was manifested against it following a preliminary studies.

## Materials and methods

### Materials

Four compounds isolated from leaves of *Mallotus oppositifolius* (Geisler) Müll. Arg. were used namely: acronyculatin S, acronyculatin T, mallotojaponin B, and lichenxanthone. Their structures had previously been elucidated by spectroscopic analyses and HRMS data [10] during our previous studies.

The antibiotics used were ciprofloxacin (Fluoroquinolones), gentamicin (Aminoglycosides), ampicillin, cloxacillin, oxacillin, and penicillin (Beta-lactam), chloramphenicol (Phenicols) and Podophyllotoxin (lignan) present in the laboratory and obtained from Sigma Aldrich.

*In vitro* antibacterial activity of the natural compounds and antibiotics was evaluated on methicillin-resistant *Staphylococcus aureus* ATCC33591 (MRSA ATCC 33591). This strain was stored at 4°C in the Laboratory of Phytobiochemistry and Medicinal Plant Studies in tubes containing glycerol-suplimented Mueller Hinton Agar. Vero cell lines ATCC CRL 158 (African green monkey kidney epithelial cells) from Centre Pasteur of Cameroon (CPC) and Raw 264.7 from Noguchi Memorial Institute for Medical Research, University of Ghana were used to evaluate the cytotoxicity.

### Methods

#### Determination of the anti-staphylococcal activity of compounds and antibiotics

*Preparation of stock solutions of compounds and antibiotics*. Stock solutions of compounds were prepared at 1 mg/mL by dissolving 1 mg of each compound in 1 mL of 10% DMSO and stored at 4°C for testing. The different antibiotics were prepared in the same conditions at 2.5 mg/mL in sterile distilled water.

*Determination of Minimum Inhibitory Concentrations (MICs) and Minimum Bactericidal Concentrations (MBCs) of natural compounds and antibiotics*. The MICs and MBCs were determined by using the 96-wells broth microdilution method as described by CLSI 2012 (protocol M07-A9) [13] with slight modifications. Thus, 160 μL of Muller Hinton Broth (MHB) was introduced into the twelve first wells and 100 μL into the rest of the wells. Then, 40 μL of each compound prepared at 1 mg/mL and antibiotic prepared at 2.5 mg/mL were introduced into the 12 previous first wells followed by a series of 6 dilutions. At the end, 100 μL of a bacterial suspension prepared at 1x10^6^ CFU/mL was introduced into each well. The final concentrations of compounds and antibiotics ranged from 100 to 1.562 μg/mL and from 250 to 0.122 μg/mL respectively. Tests were carried out alongside negative control which consisted of medium and bacterial suspension as well as sterility control wells which had medium only. The microplates were covered and incubated at 37°C for 24 hours. At the end of the incubation period, 20 μl of 0.2 mg/mL ρ-iodonitrotetrazolium chloride (INT; Sigma Aldrich) was used as a revelator. The lowest concentration without any color change (from yellow to pink) was considered as the MIC. For Minimum Bactericidal Concentrations (MBCs) determination, 25 μL aliquots from corresponding inhibitory wells, that did not receive INT, were transferred into wells containing 175 μL of sample-free broth medium. After 48 h of incubation at 37°C, MBC was revealed as previously described using INT. All the experiments were performed in triplicate.

*Evaluation of the anti-staphylococcal activity of the combination of phloroglucinol with an antibiotic*. ***Preparation of the intermediate plate*.** The intermediate plates were prepared from a stock solution of the best active compound (mallotojaponin B) at a concentration of 50 μg/mL and the less active antibiotic (chloramphenicol) at a concentration of 1mg/mL in MHB. Different volumes of the compound and antibiotic solution were each introduced into two different microplates to obtain 4MICs (50 μg/mL) in the wells of the first row of the microplate. This was followed by serial two-folds dilution along the columns (1 to 8) to obtain a concentration range of 4MIC (50 μg/mL) to MIC/32 (0.3906 μg/mL) for compound and antibiotic which were all adjusted to 100 μL.

#### Determination of Fractional Minimum Inhibitory Concentration Indices (FICI)

The determination of FICI of the compound with antibiotic was evaluated by the checkerboard dilution method [11]. For this, twenty-five microliters (25 μL) of the different concentrations from the intermediate plates were permutatively added into the wells of the tests plate to have the compound and antibiotic in the same well at all the different possible concentrations of the compound (MIC (12.5 μg/mL) in well 1 to MIC/128 (0.097 μg/mL) in well 8) and antibiotic (MIC (250 μg/mL) in well A to MIC/128 (1.953 μg/mL) in well H) followed by the addition of 50 μL of bacterial suspension (1 ×10^6^ CFU/mL) to have the final volume of 100 μL in each test well. MHB mixed with bacterial suspension served as the negative control while MHB alone was the sterility control. The tests were performed in triplicate and plates were incubated at 37°C for 24 h. At the end of the incubation time, INT was used as a revelator as described previously. Subsequently, the Minimum Fractional Inhibitory Concentration (FICI) of the different couples of concentrations was calculated to determine the type of interaction using the following formula:

FICI=FICA+FICB

$$FICA=\frac{MICA\;com}{MICA}$$

and

$$FICB=\frac{MICB\;com}{MICB}$$

A = compound and B = antibiotic in combination

Interpretation of the type of interaction was done according to the values of FICI:

FICI ≤ 0.5, the interaction was synergistic;

0.5< FICI ≤ 1.0 it additive;

1.0 < FICI≤4.0 means no interaction (non-differential);

FICI > 4.0, the interaction was antagonistic.

**Fig 1:**
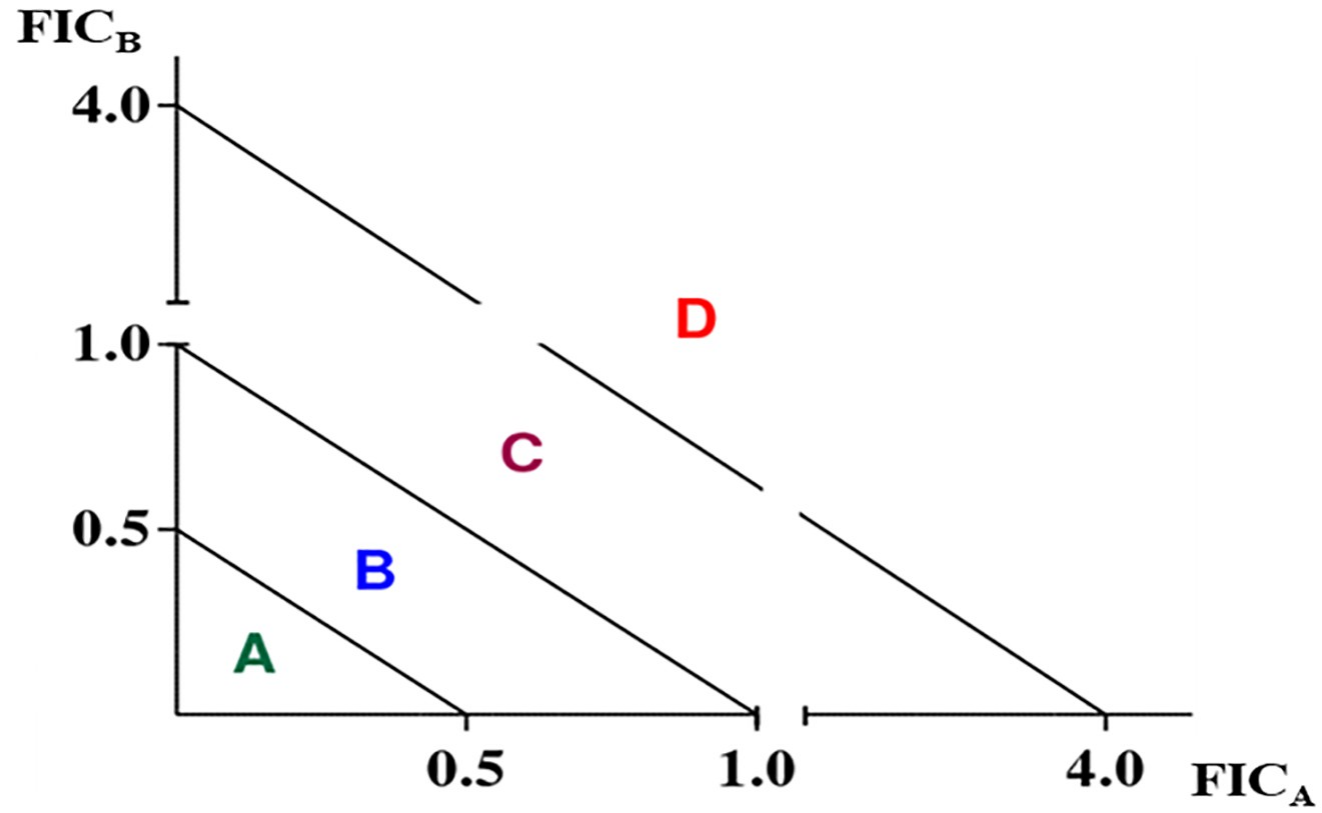
The diagonal lines for the interpretation of isobolograms in the interaction study between two bioactive substances. A: Synergy, B: Additivity, C: Indifference, D: Antagonism.

The tests were performed in triplicate and in addition to the arithmetic interpretation of the combination, the type of interaction involved was appreciated geometrically based on the use of isobolograms; obtained by representing the FIC values of one of the products (FICa) in combination with that of the other (FICb). (Fig 1).

The antibiotic and the pairs of combinations that showed a synergistic interaction were selected for their cytotoxicity evaluation.

#### Cytotoxicity evaluation of the antibiotic and synergistic combinations

The evaluation of the cytotoxicity of the least active antibiotic and synergistic combinations was done using Vero and Raw cell lines by the resazurin-based colorimetric method as described by Zwolak and Cho-k, [14]. Briefly, in a 96-well microplate, 100 μL of a cell suspension at 10^4^ cells/well was introduced into all the wells and incubated for 24h at 37°C for adhesion. At the end of the incubation period, the culture medium containing the non-adherent cells was removed and 90 μL of new medium and 10 μL of each test solution were added to the test wells, and the plates were then incubated for 48h at 37°C, 5% CO_2_. The wells corresponding to the positive control, negative control, and sterility control contained 10μM podophyllotoxin, cells without inhibitor, and culture medium respectively. After incubation, 10 μL of resazurin solution (0.15 mg/mL) was introduced into each well and the plate was incubated for 4h at 37°C, 5% CO_2_. At the end of this incubation period, fluorescence was measured at an excitation and emission wavelength of 530 and 590 nm respectively using a TECAN microtiter plate reader (Infinite M200). From the optical densities obtained, the percentages of cell inhibition were calculated with Microsoft Excel using the following formula

$$\%\;inhibition=\frac{\left(Ac-At\right)}{\left(Ac\right)}X\;100$$

Where, Ac = Absorbance of the negative control, At = Absorbance of the Test.

#### Modes of antibacterial action of the compound and synergistic combinations

*Evaluation of the time kill kinetics*. The time-kill kinetics of our test solutions at different concentrations was evaluated using the method described by Klepser *et al*. in 1998 [15] with slight modifications. Briefly, in 96-well microplates, 100 μL of Muller Hinton broth containing the compound (50 μg/mL) was introduced into each well followed by 3 series of geometric dilutions of order 2, and the different combinations were introduced in the corresponding wells. Then 100 μL of MRSA suspension (1x10^6^ cells/mL) was added to each well except the sterility control wells. The plates were covered and incubated at 37°C at different times (0h, 2h, 4h, 6h, 8h, 12h, and 24h). After each incubation period, ODs were measured at 620 nm using the TECAN infinite M200 plate reader. Gentamicin (1.25 μg/mL) was used as a positive control and the tests were performed in triplicate. The optical densities versus incubation time curves were plotted and these curves permitted the determination of the time for the onset of activity and the optimum activity for the combinations and the compound as well as their bactericidal effects.

*Nucleotide leakage*. This assay was performed according to the protocol described by Tang *et al*. [16]. The tests were performed in triplicate using Eppendorf tubes. Overnight bacterial cultures were washed twice in PBS (10mM), then re-suspended in the same buffer (10mM PBS) and adjusted to the 0.5 McFarland turbidity standard. Subsequently, the bacterial suspension was introduced into 1mL of 10mM PBS containing different concentrations of mallotojaponin B (25, 12.5, and 6.25 μg/mL) and combinations. After that, the whole set was incubated at 37°C at different times (0h, 2h, 4h, 6h, 8h, and 12h). After each incubation time, the solutions were centrifuged at 10 000 rpm for 10 min. The supernatant was collected and the OD was measured at 260 nm using the TECAN infinite M200 plate reader. The Optical Densities were used to plot the OD versus time curve which highlights the leakage of nucleic acids (an increase of OD with time) as an indicator of membrane damage of the different samples.

*Evaluation of the loss of salt tolerance*. The ability of MRSA ATCC33591 to form colonies in the presence of active compounds and combinations on Muller Hinton Agar (MHA) supplemented with NaCl was evaluated according to the protocol previously used by Etame *et al*. [17]. A preliminary test was performed by inoculating the strain on MHA medium supplemented with NaCl at different concentrations ranging from 1% to 10% and incubated at 37°C for 24h. At the end of the incubation time, the colonies were counted and the highest NaCl concentrations tolerated by the bacteria were selected for the next assay. Subsequently, the bacterial suspension was prepared at 5x10^5^ CFU/mL and treated with a solution of the compound at 25, 12.5, and 6.25 μg/mL and combinations. The microplates were incubated at 37°C for 1h and then, subcultured on MHA supplemented with NaCl at the highest tolerable concentrations and Petri dishes were incubated at 37°C for 24h. After the incubation period, colonies were counted and the curve of colony count versus NaCl and compound concentration was plotted.

#### Anti-biofilm activities of the compound and synergistic combinations on MRSA ATCC33591

*Quantification of biofilm biomass*. For biofilm production, the bacterial suspension was prepared from the overnight bacterial culture and adjusted at 1×10^6^ CFU/mL in a different medium. The media used were MHB, Tryptic Soy (TS) broth, and Brain Heart Infusion (BHI) broth supplemented with glucose at 1% and 2%. After that, 200 μL of bacterial suspension was added in each well of a 96-well flat-bottom microplate. The negative control consisted of medium and bacterial suspension, and the sterility control contained only medium. The microplates were covered and then incubated at 37°C for 24 and 48 hours. The biofilm biomass was measured by crystal violet staining according to the protocol described by Stepanović *et al*., [18] with slight modifications. After incubation, the microplates were emptied and 200 μL of 99% ethanol was added to each well and the microplates were incubated for 15 mins. The wells were subsequently washed twice with PBS and then 200 μL of 0.1% crystal violet (Sigma-Aldrich) was added to each well. The plate was incubated at room temperature for 20 mins after which the crystal violet residues were removed and the wells were washed twice with PBS. Then, 200 μL of 33% sulfuric acid was introduced into each well and the absorbance was read at 570 nm using the TECAN plate reader (Infinite M200). The results obtained were used to select the best medium that permitted highest biofilm formation.

*Determination of the Median Minimum Biofilm Inhibitory Concentration (MBIC*_*50*_*) and the Median Minimum Biofilm Eradicating Concentration (MBEC*_*50*_*)*. MBIC_50_ and MBEC_50_ were determined by the liquid microdilution method in 96-well flat-bottom microplates using the protocol described by CLSI [13]. For this, 100 μL of Muller Hinton broth supplemented with 2% glucose containing the compound (100 μg/mL) was introduced into the first wells followed by a serial two-fold dilution. Subsequently, 100 μL of the combinations were introduced into the corresponding wells. Then 100 μL of a bacterial suspension was added to all wells except those of the sterility control followed by incubation at 37°C for 24h. The final bacterial load in the wells was 1×10^6^ CFU/mL. The final concentrations of ciprofloxacin (positive control) and compound ranged from 50 μg/mL to 12.5 μg/mL. After incubation, the microplate was treated as described previously and the absorbance was measured at 570 nm using a TECAN brand plate reader (Infinite M200).

The MBEC_50_ was determined in the same conditions with the only difference being that the biofilm was first formed in the Muller Hinton broth supplemented with glucose at 2% during 24 hours. The compound and combinations were prepared as described above and introduced into the plates containing the biofilms. Then the plate was incubated in the same conditions and treated as previously described. The percentages of inhibition and eradication of the biofilm were calculated using the following formula:

$$\%inhibition/eradication=\frac{\left(DOc-DOt\right)}{\left(DOc\right)}X\;100$$

Where ODc = Optical Density of the negative control, ODt = Optical Density test

MBIC_50_ and MBEC_50_ were defined as the minimal concentration that inhibited 50% of biofilm formation and the minimal concentration that eradicated 50% of the biofilms formed respectively.

### Statistical analysis

Statistical analysis was carried out using the ’One Way Analysis of Variance’ (ANOVA) with the SPSS (Statistic Package for Social Science) software version 16.0 for analyzable data. The results were expressed as Mean ± Standard Deviation. The differences between the means were compared by the Waller-Duncan test at 95% confidence level (p ≤0.05). Excel software was used to plot the graphs.

## Results

### The anti-staphylococcal activity of natural compounds and antibiotics

Table 1 presents the minimal inhibitory concentrations and minimal bactericidal concentrations of 4 phloroglucinols, compounds isolated from *Mallotus oppositifolius*, and 6 antibiotics used for the treatment of Staphylococcal infections.

**Table 1:** Minimal Inhibitory Concentrations (MICs) and Minimal Bactericidal Concentrations (MBCs) of compounds and antibiotics on MRSA ATCC 33591.

Inhibition parameters				
Natural compounds and antibiotics		MIC (μg/mL)	MBC (μg/mL)	MBC/MIC
Natural Compounds	acronyculatin S	>100	/	/
	acronyculatin T	>100	/	/
	mallotojaponin B	12,5	50	4
	lichenxanthone	>100	/	/
Antibiotics	ampicillin	0.122	Nt	/
	cloxacillin	0.488	Nt	/
	oxacillin	0.122	Nt	/
	chloramphenicol	250	Nt	/
	penicillin	0.488	Nt	/
	gentamycin	0.976	Nt	/

/: not determined; nt: not tested; MRSA: *Staphylococcus aureus* Methicilin Resistant ATCC33591

This table shows that amongst the four natural compounds tested (acronyculatin S, acronyculatin T, mallotojaponin B, and lichenxanthone), only mallotojaponin B showed inhibitory activity against MRSA ATCC 33591 with MIC and MBC values of 12.5 μg/mL and 50 μg/mL respectively; while the MICs of antibiotics ranged from 0.112 to 250 μg/mL. According to the classification criteria defined by Walsh *et al* [19], a strain is considered ‘moderately resistant’ towards an antibiotic when the MIC ≥ 8 μg/mL and ‘clinically resistant’ when the MIC is greater than 32 μg/mL. Therefore, of the five antibiotics tested the MRSA strain was resistant only to chloramphenicol (MIC 250 μg/mL) and susceptible to ampicillin, cloxacillin, oxacillin, and penicillin.

### Interaction between mallotojaponin B and chloramphenicol and cytotoxicity of combinations

#### Interaction between mallotojaponin B and chloramphenicol

Table 2 presents the interactions between the natural compound, mallotojaponin B (most active) and chloramphenicol (less active), through their fractional minimum inhibitory concentration indices, against MRSA ATCC 33591.

**Table 2:** Fractional Minimum inhibitory concentration indices (FICI) of different combinations of mallotojaponin B and chloramphenicol vis-à-vis MRSA ATCC 33591.

Corresponding Wells MICs	Conc of C6 (μg/mL)	Conc of CPL (μg/mL)	FIC C6	FIC CPL	FICI
E1	0.781	250	0.062	1	1.062(I)
E2	0.781	125	0.062	0.5	0.562(A)
E3	0.781	62.5	0.062	0.25	0.312(S)
E4	0.781	31.25	0.062	0.125	0.187(S)
E5	0.781	15.625	0.062	0.063	0.124(S)
E6	0.781	7.812	0.062	0.031	0.093(S)
D7	1.562	3.9	0.125	0.016	0.14(S)
E8	0.781	1.95	0.062	0.008	0.069(S)
C7	3.125	3.9	0.25	0.016	0.265(S)
B8	6.25	1.95	0.5	0.008	0.507(A)
A8	12.5	1.95	1	0.008	1.007(I)
**Mean of FICI**					**0.393 (S)**

E1; E2; E3; E4; E5; E6; E8; D7; C7; B8; A8: Wells representing the different pairs of concentrations considered as MICs; Conc: Concentration in ug/mL; CPL: chloramphenicol; C6: mallotojaponin B; FIC: Fractional Minimum Inhibitory Concentration; FICI: Fractional Minimum Inhibitory Concentration Index MRSA ATCC 33591: Methicillin Resistant *Staphylococcus Aureus* ATCC 33591; S = Synergy; A = Additive I = Indifference (no interaction)

**Fig 2:**
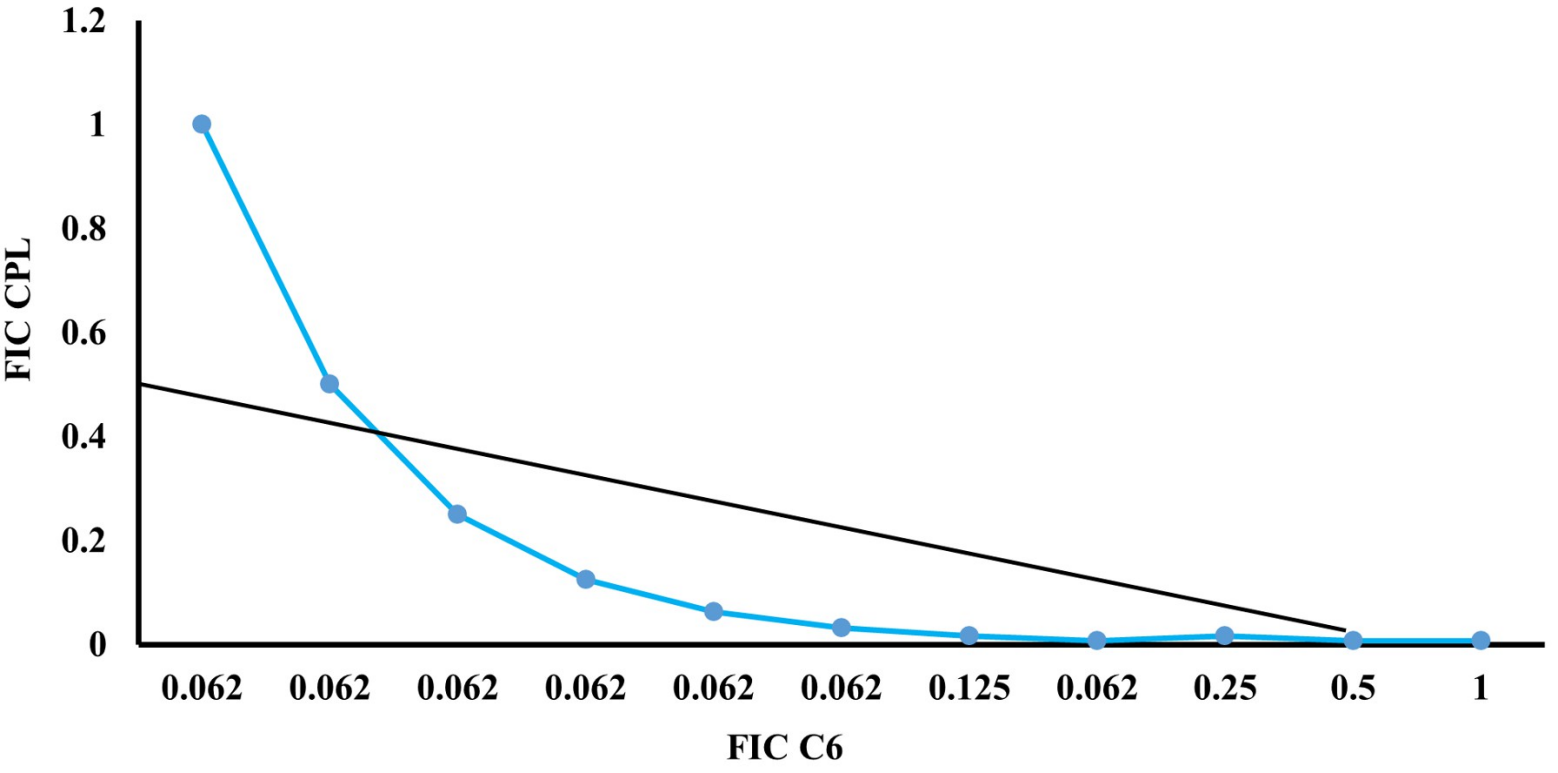
Isobologram representing the synergistic interaction between mallotojaponin B and chloramphenicol on MRSA ATCC 33591.

Table 2 shows that the combination of mallotojaponin B and chloramphenicol leads to a mutual potentiation of their activities against MRSA. This is justified by the fact that the MICs reduced from 12.5 μg/mL (MIC) to 0.781 μg/mL (1/16 MIC) and from 250 μg/mL (MIC) to 1.95 μg/mL (1/128 MIC) for mallotojaponin B and chloramphenicol, respectively. The mean FICI of 0.393 suggests that the combination between mallotojaponin B and chloramphenicol is synergistic [12]. This result of synergism is confirmed by Fig 2, which is an isobologram derived from the representation of the FIC of chloramphenicol versus the FIC of mallotojaponin B which turns out to be a convex curve whose point cloud passes below the coordinate line (0.5–0.5).

### Percentage inhibition of Vero and Raw cells growth by the synergistic combinations

**Fig 3:**
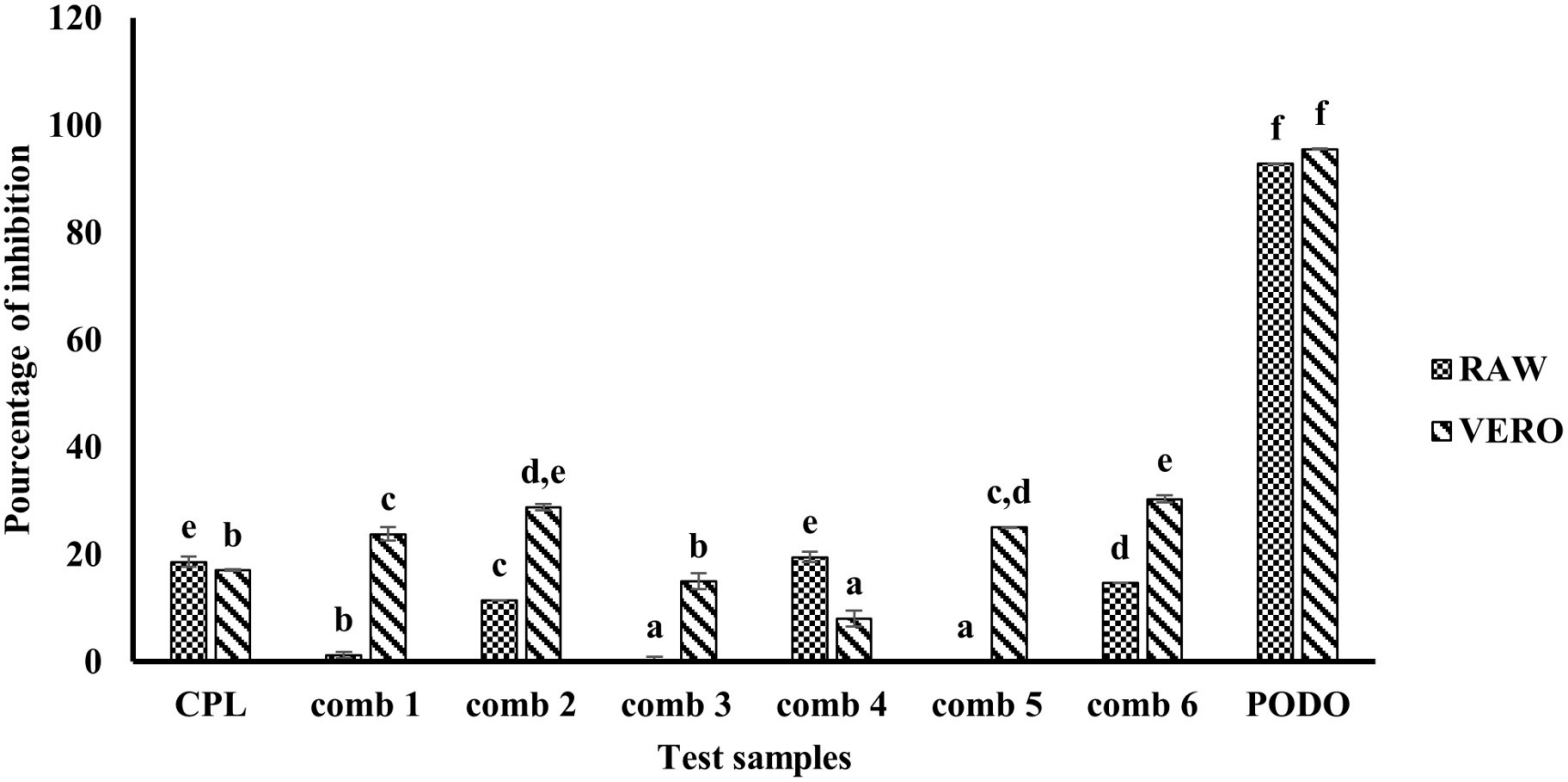
Percentage of inhibition of synergistic combinations and chloramphenicol on Vero and Raw cells. CPL: chloramphenicol; Comb: combination; Comb 1: 0.781 μg/mL of mallotojaponin B and 1.95 μg/mL of chloramphenicol; Comb 2: 0.781 μg/mL of mallotojaponin B and 7.812 μg/mL of chloramphenicol; Comb 3: 1.562 μg/mL of mallotojaponin B and 3.9 μg/mL of chloramphenicol; Comb 4: 0.781 μg/mL of mallotojaponin B and 15.625 μg/mL of chloramphenicol; Comb 5: 0.781 μg/mL of mallotojaponin B and 31.25 μg/mL of chloramphenicol; Comb 6: 3.125 μg/mL of mallotojaponin B and 3.9 μg/mL of chloramphenicol; PODO: Podophyllotoxin; For the same cell lines, bars with the same letter show that there is no significant difference between the different treatments at p ≤0.05.

To evaluate the safety of the synergistic combinations we determined their percentage of growth inhibition on two normal cell lines Vero and Raw as presented in Fig 3.

The percentage of inhibition was evaluated on six synergistic combinations: combination1 (FICI = 0.069), combination2 (FICI = 0.093), combination3 (FICI = 0.140), combination4 (FICI = 0.124), combination5 (FICI = 0.0187), combination6 (FICI = 0.265) and the result is presented in Fig 3. This figure shows that the percentage of inhibition varies from 8.065% to 30.328% on the Vero cells and 0.00% to 19.50% on the Raw cells. According to the classification criteria reported by Kazakova *et al*. [20], a substance is considered non-cytotoxic if the percentage of inhibition is less than or equal to 32%, therefore, no selected synergistic combination was cytotoxic.

### Modes of action of mallotojaponin B and the synergetic combinations

#### Time kills kinetics

**Fig 4:**
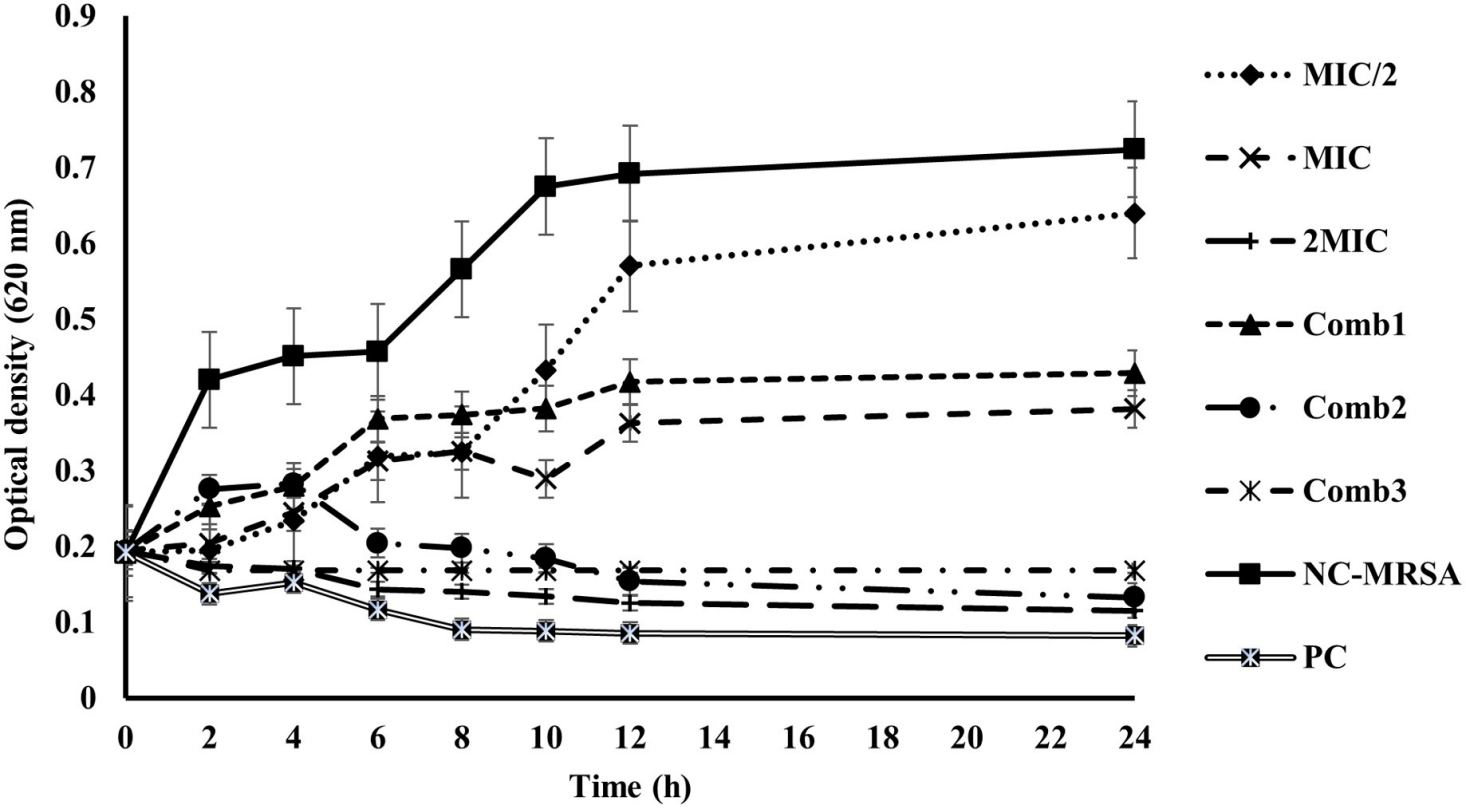
Growth curves of MRSA following exposure to various concentrations of mallotojaponin B and synergistic combinations. MIC: Minimum Inhibitory Concentration; PC: positive control (Ciprofloxacin); NC: negative control; Comb: combination; Comb 1: 0.781 μg/mL of mallotojaponin B and 1.95 μg/mL of chloramphenicol; Comb 2: 0.781 μg/mL of mallotojaponin B and 7.812 μg/mL of chloramphenicol; Comb 3: 1.562 μg/mL of mallotojaponin B and 3.9 μg/mL of chloramphenicol.

Fig 4 shows the growth of MRSA ATCC 33591 at different times in the presence of mallotojaponin B at different concentrations and synergetic combinations 1, 2, and 3.

Fig 4 shows that in comparison to the negative control and positive control, mallotojaponin B and synergetic combination at different concentrations showed an inhibitory effect on bacterial growth. mallotojaponin B at 6.25 μg/mL (1/2 MIC) and 12.5 μg/mL (MIC) and Combination 1 have a weak inhibition of bacterial growth. Moreover, from 8 hours of mallotojaponin B at 25 μg/mL (2MIC), Combinations 2 and 3 showed the ability to inhibit bacterial growth at a rate that was not significantly different (P˃0.05) from that of Ciprofloxacin used as positive control and they have a bactericidal effect against MRSA ATCC 33591.

#### Nucleotide leakage

**Fig 5:**
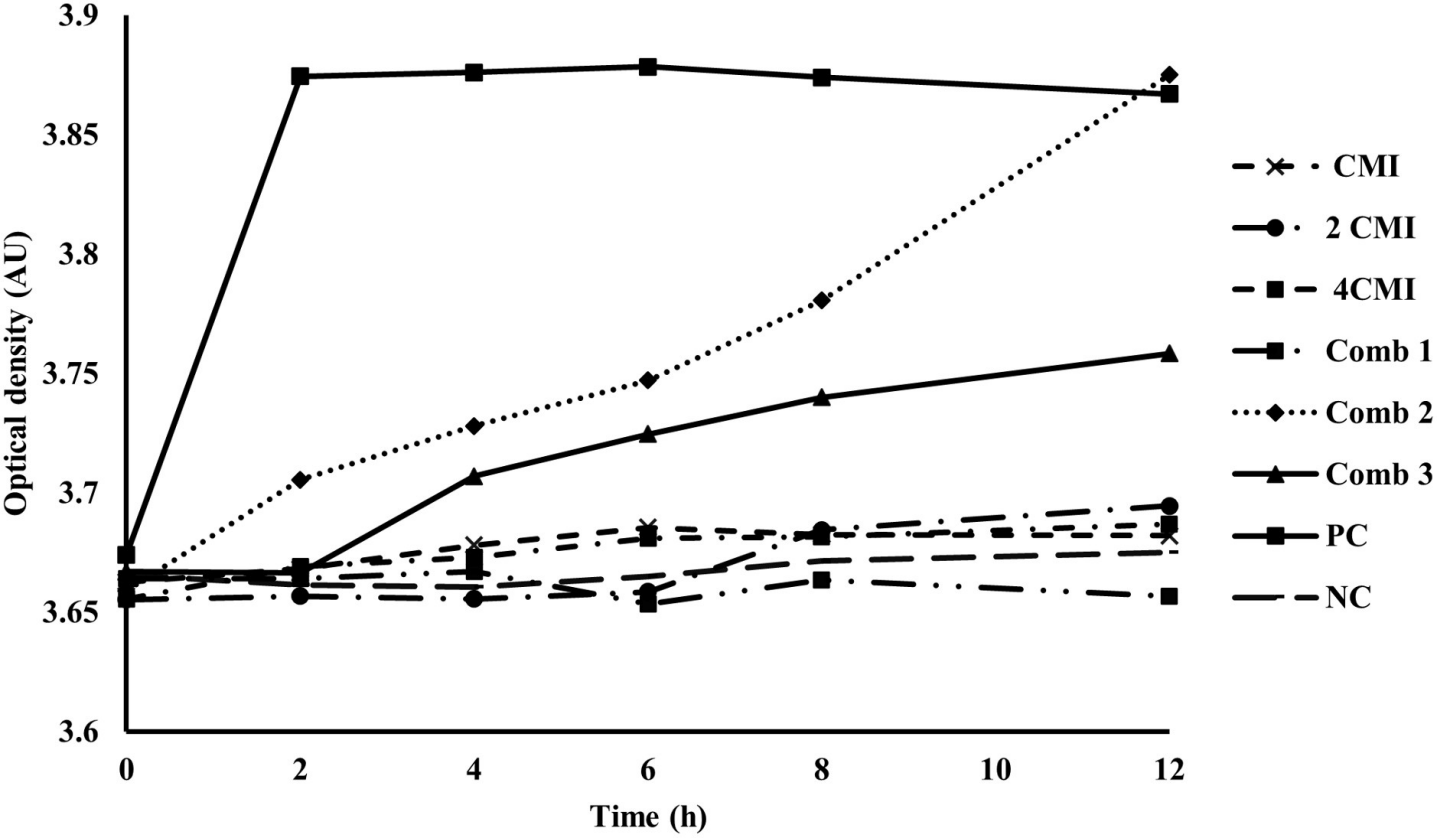
Effect of the mallotojaponin B and the different combinations on nucleotide leakage MRSA ATCC 33591. MIC: Minimum Inhibitory Concentration; PC: positive control (TritonX); NC: negative control; Comb: combination; Comb 1: 0.781 μg/mL of mallotojaponin B and 1.95 μg/mL of chloramphenicol; Comb 2: 0.781 μg/mL of mallotojaponin B and 7.812 μg/mL of chloramphenicol; Comb 3: 1.562 μg/mL of mallotojaponin B and 3.9 μg/mL of chloramphenicol.

Fig 5 shows the quantification of nucleic acids (expressed in optical density) released under the potential effect of different concentrations of mallotojaponin B and combinations.

Fig 5 shows in comparison to the positive control (Triton X 0.1N) that combinations 2 and 3 significantly cause the release of nucleic acids, thus acting by inducing membrane permeabilization. This is shown by a progressive increase in optical densities as a function of time which are proportional to nucleic acid concentrations. mallotojaponin B at different concentrations (12.5, 25, and 50 μg/mL), combination 1 did not significantly (p˃0.05) cause the release of nucleic acids.

#### Loss of salt tolerance

**Fig 6:**
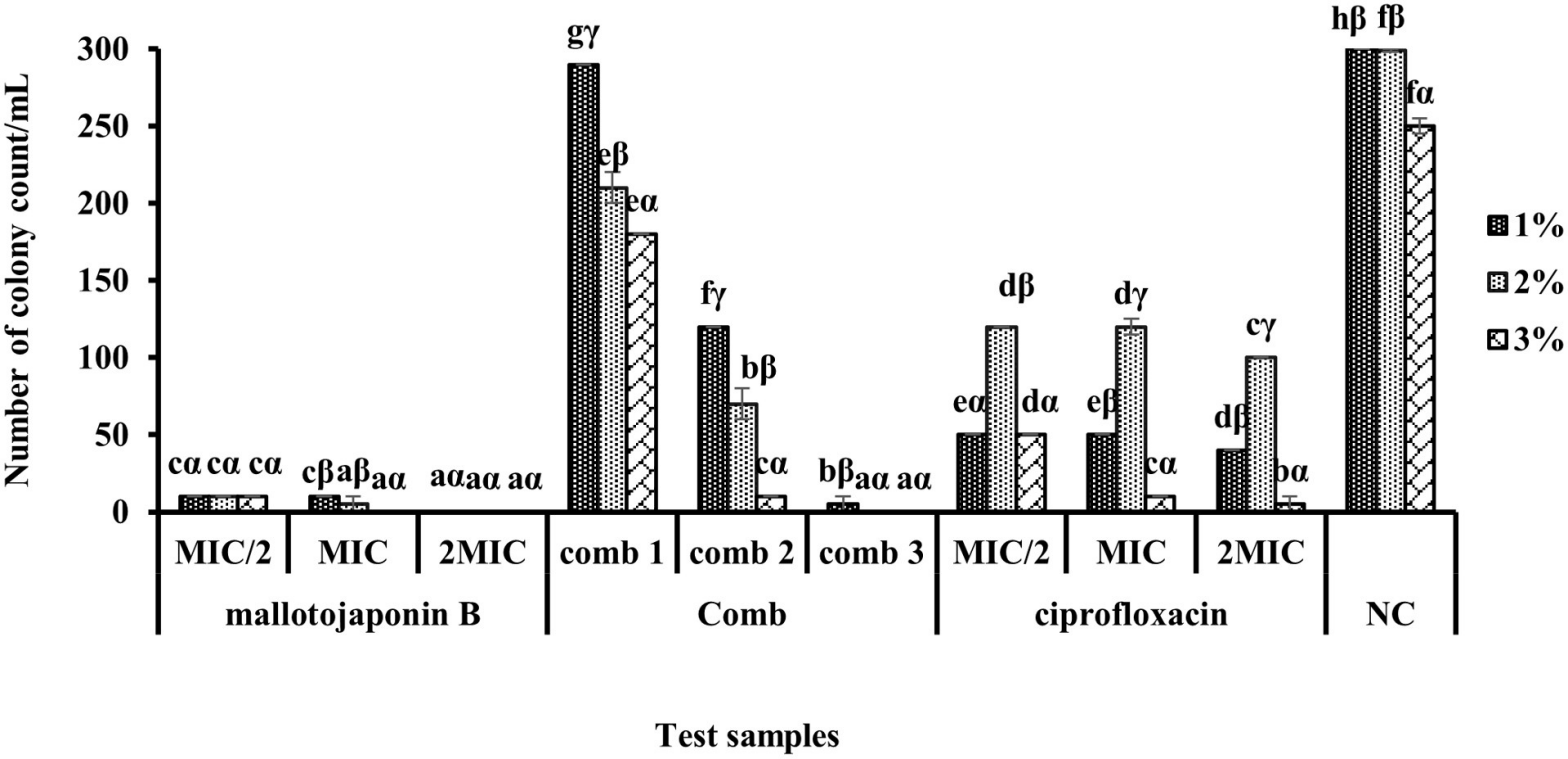
Variation in the number of *S*. *aureus* colonies as a function of extract and NaCl concentration. MIC: Minimum Inhibitory Concentration; PC: positive control; NC: negative control; Comb: Combination; Comb 1: 0.781 μg/ml of mallotojaponin B and 1.95 μg/mL of chloramphenicol; Comb 2: 0.781 μg/mL of mallotojaponin B and 7.812 μg/mL of chloramphenicol; Comb 3: 1.562 μg/mL of mallotojaponin B and 3.9 μg/mL of chloramphenicol; For each salt concentration, histograms carrying the same letters are not significantly different (p˃0.05); while for each test sample, histograms with same Greek alphabets are not significantly different, Waller Duncan test.

The loss of salt tolerance of MRSA in the presence of mallotojaponin B at different concentrations and combinations in MHA medium supplemented with NaCl at different concentrations is illustrated in Fig 6.

From Fig 6, it appears that in general, the loss in salt tolerance is concentration-dependent and characterized by a decrease in the number of colonies formed as the concentration of NaCl and test substance increase. Thus, lower salt tolerance is observed when the concentration of mallotojaponin B is 12.5 μg/mL and 25 μg/mL as well as combination 3 and the salt concentration is 3%. This results in the eradication of bacterial growth (0 CFU) compared to the negative control (250 CFU). At MIC/2 concentration of 6.25 μg/mL, the number of colonies decreased (10 CFU) compared to the negative control (250 CFU), which justifies the effect of mallotojaponin B on the outer membrane preventing the expulsion of salts from the cell.

#### Inhibition and eradication of biofilm

**Fig 7:**
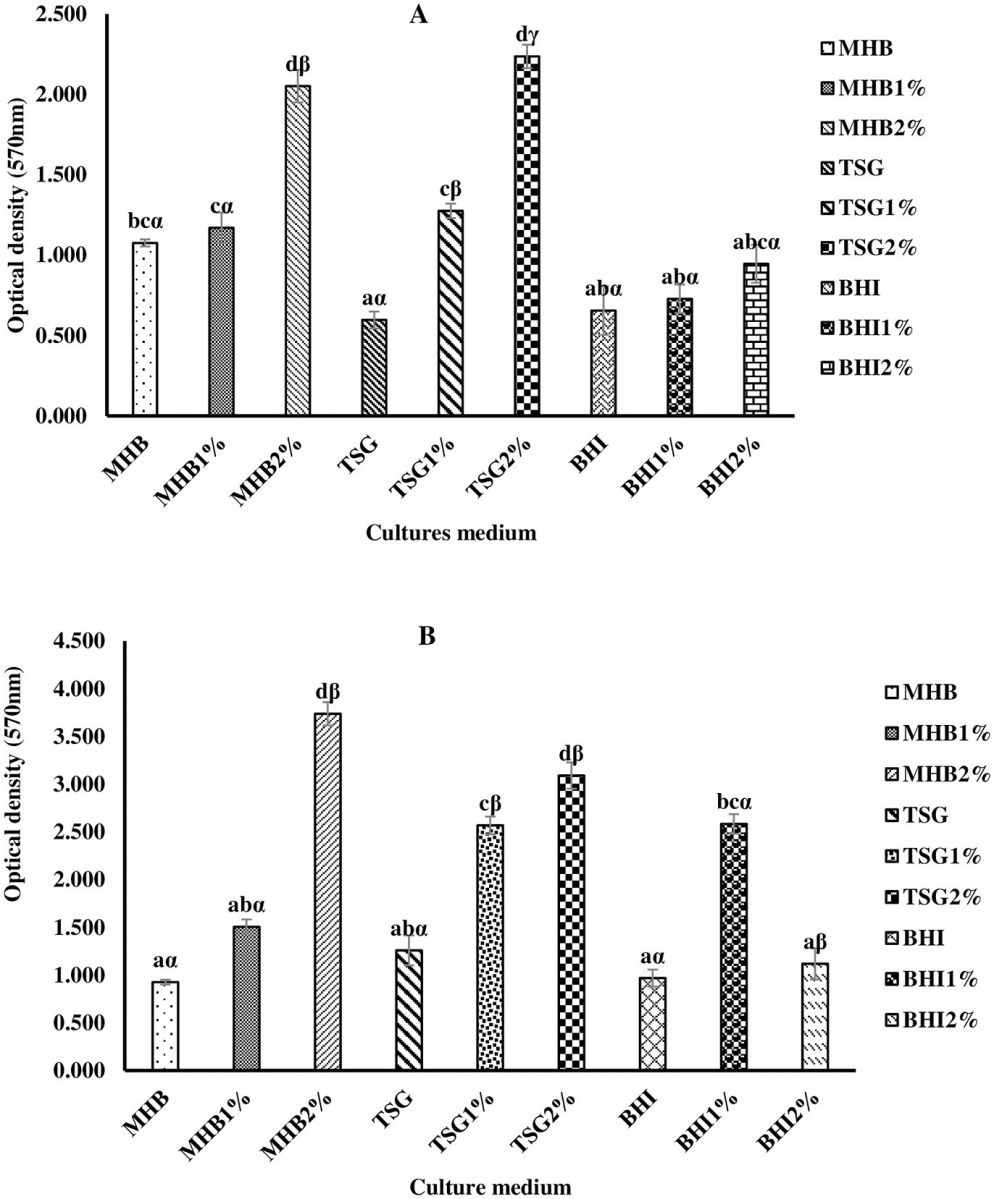
(A and B). Biomass of biofilms produced by MRSA ATCC 33591 in different cultures medium after 24 and 48hrs of incubation. MHB: Muller Hinton Broth; MHB 1%: Muller Hinton Broth with Glucose 1%; MHB 2%: Muller Hinton Broth with Glucose 2%; TSG 1%: Tryptic Soy with Glucose 1%; TSG2%: Tryptic Soy with Glucose 2%; BHI: Brain Heart Infusion; BHI 1%: Brain Heart Infusion with Glucose 1%; BHI 2%: Brain Heart Infusion with Glucose 2%. Histograms with the same letters are not significantly different (p ˃0.05), while those carrying the same Greek alphabet are not significantly different for the same medium.

**Fig 8:**
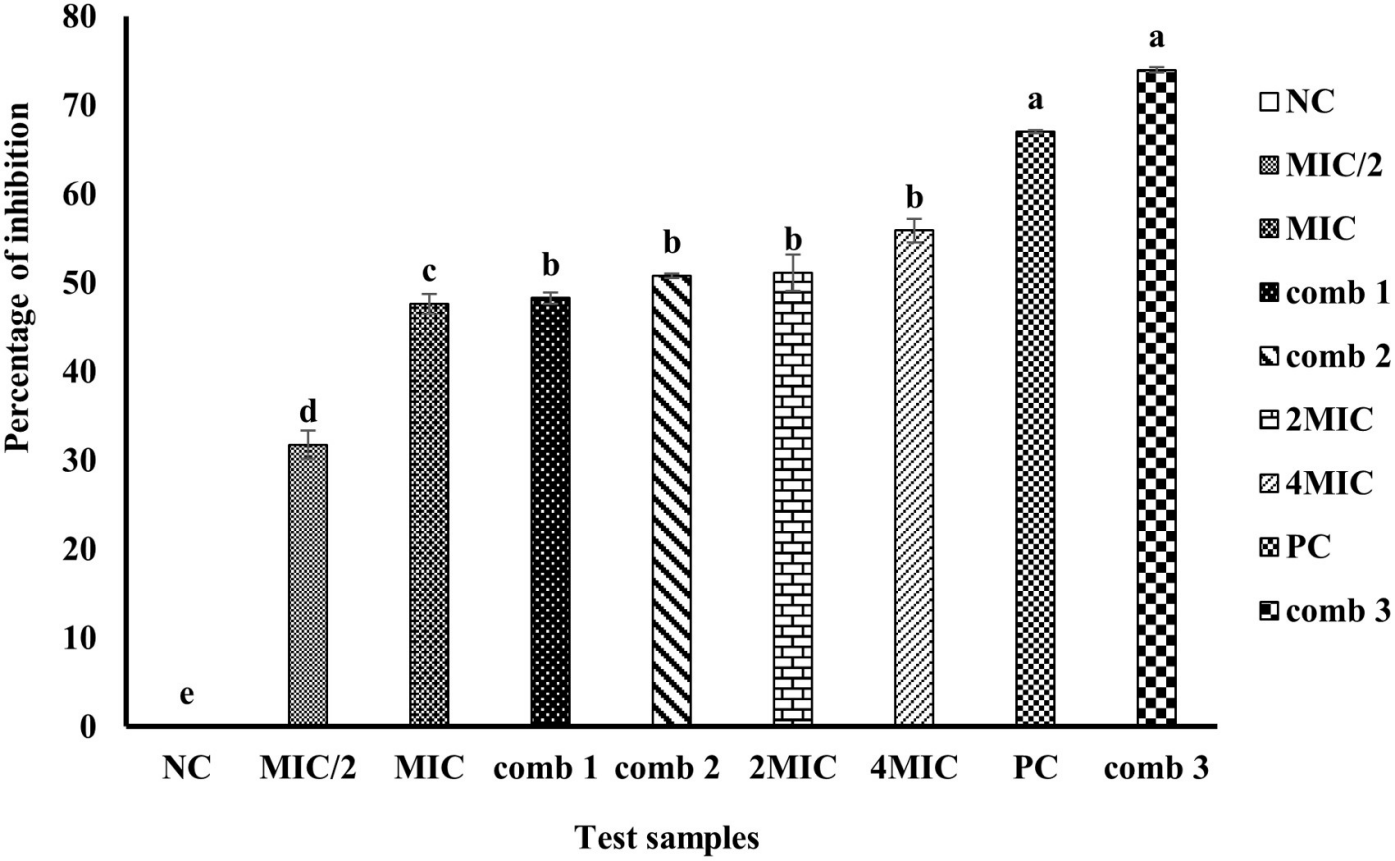
Percentage of inhibition of biofilm of MRSA ATCC 33591 in MHB 2%. CP: Ciprofloxacin; Comb: Combination; Comb 1: 0.781 μg/mL of mallotojaponin B and 1.95 μg/mL of chloramphenicol; Comb 2: 0.781 μg/mL of mallotojaponin B and 7.812 μg/mL of chloramphenicol; Comb 3: 1.562 μg/mL of mallotojaponin B and 3.9 μg/mL of chloramphenicol; MIC: Minimum Inhibitory Concentration; NC: Negative Control. Histograms with the same letters are not significantly different (p˃0.05).

*Quantification of biomass of biofilms*. Figs 7A and 7B and 8 represent the quantity of biomass of biofilm in different culture media using Muller Hinton broth, Tryptic soy, and Brain Heart Infusion supplemented with glucose at 1% and 2% after 24 (A) and 48 (B) hours.

From Fig 7A and 7B, MHB and TSG broths better promote the production of biofilm. Furthermore, these Figures show that the amount of biofilm formed is doubled when the medium is supplemented with 2% glucose. Similarly, after 48 h of incubation, the amount of biofilm formed is double relative to 24 h. There was no significant difference (P˃0.05) between the amount of biofilm formed in MH broth and TSG supplemented with glucose at 2%. Thus, the MH broth was chosen for the inhibition and eradication of biofilm.

*Inhibition and eradication of biofilm*. ***Biofilm inhibition*.** Fig 8 presents the inhibition of biofilms by the synergetic combinations (1, 2, and 3) and mallotojaponin B (6.25, 12.5, and 25 μg/mL).

Fig 8 shows that the synergistic combinations and mallotojaponin B inhibit the formation of biofilm. The biofilm-inhibition capacity of combination 3 was significantly greater (P≤0.05) than that of combination 1, combination 2, mallotojaponin B at 50 μg/mL and 25 μg/mL; and comparable to that of Ciprofloxacin used as the positive control. The different combinations and mallotojaponin B inhibited biofilm formation with inhibition percentages ranging from 31.75 to 73.97%. The MBIC_50_ of mallotojaponin corresponds to 25 μg/mL. Combinations 3 and 2 inhibited, respectively 73.97% and 50% of the biofilm produced by MRSA ATCC 33591.

**Fig 9:**
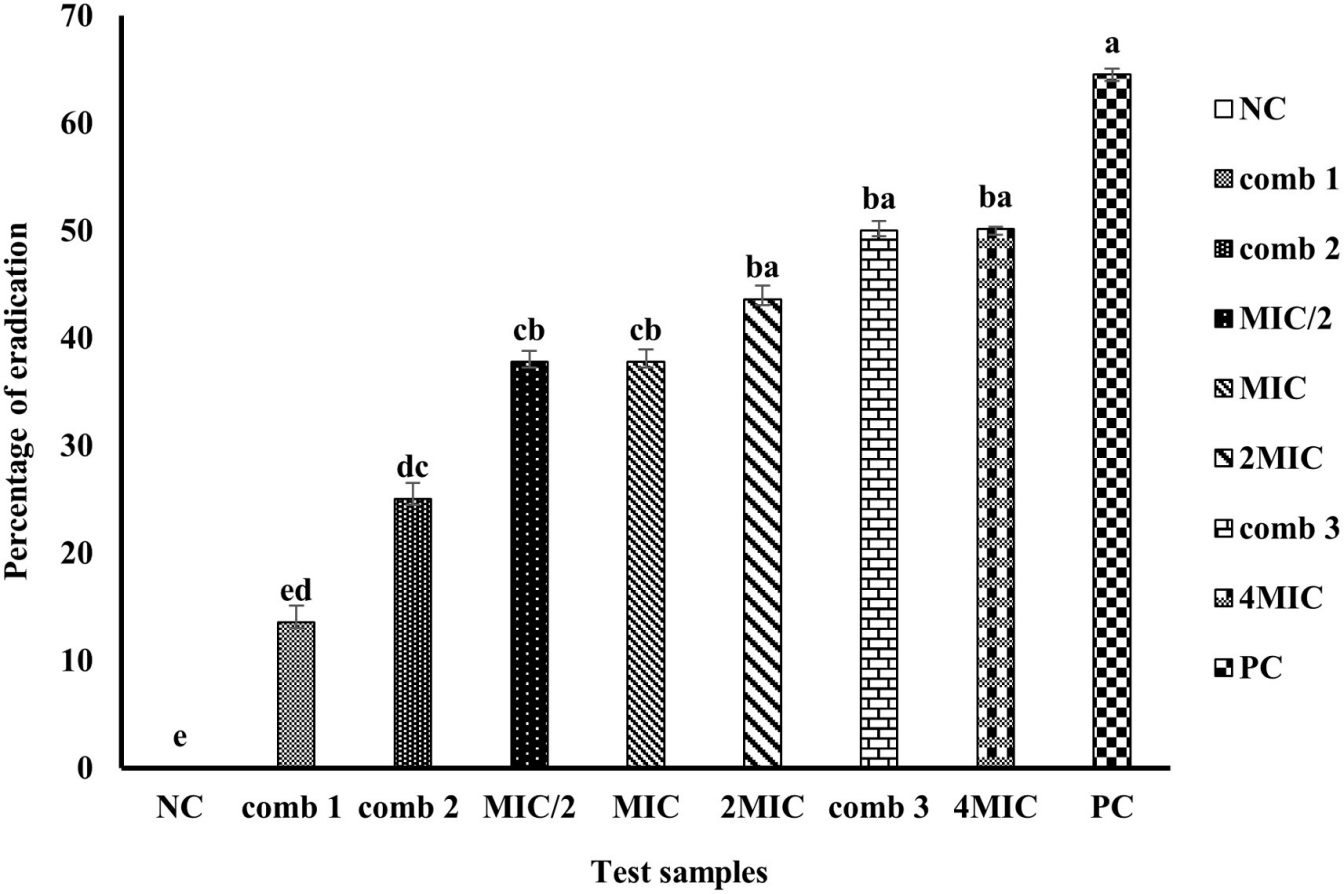
Biofilms eradication percentage of mallotojaponin B at different concentrations and combinations. PC: Ciprofloxacin; Comb: Combination; Comb 1: 0.781 μg/mL of mallotojaponin B and 1.95 μg/mL of chloramphenicol; Comb 2: 0.781 μg/mL of mallotojaponin B and 7.812 μg/mL of chloramphenicol; Comb 3: 1.562 μg/mL of mallotojaponin B and 3.9 μg/mL of chloramphenicol; MIC: Minimum Inhibitory Concentration; NC: Negative Control. Histograms with the same letters are not significantly different at p ≤0.05.

***Biofilms eradication capacities of samples*.** Fig 9 presents the percentage of eradication of biofilms formed by the synergistic combinations (1, 2, and 3) and mallotojaponin B at different concentrations (6.25, 12.5, and 25 μg/mL).

Fig 9 shows that the percentages of biofilm eradication varied between 13.56 and 64.50. Combinations 1, 2, and 3 and mallotojaponin B eradicate the biofilms formed in a concentration-dependent manner. There was a significantly higher (p≤0.05) biofilm-eradication potential induced by combination 3, mallotojaponin B at 50 μg/mL and 25 μg/mL relative to the activities of combinations 1, 2, and mallotojaponin B at 12.5 μg/mL and 6.25 μg/mL. These results suggest that combination 3, mallotojaponin B at 50 and 25 μg/mL has a more pronounced eradicative effect. The MBEC_50_ of mallotojaponin B was 50 μg/mL. Combination 3 eradicated 50% of the biofilm formed.

## Discussion

Methicillin-resistant *Staphylococcus aureus* (MRSA) has been recognized as a high-priority pathogen for research and development of new antibiotics by WHO [7] given its severe socio-economic impact worldwide. Available antibiotics for the treatment of MRSA infections have many limitations like adverse side effects and the development of resistance to standard drugs. Thus, to contribute to the development of new drugs, natural compounds isolated from medicinal plants would be an alternative to designing the new drugs against MRSA infection. This work was carried out to identify and define a potential synergistic combination between chloramphenicol and a potent previously demonstrated antibacterial phloroglucinol isolated from *Mallotus oppositifolius* [10] against MRSA.

The compounds acronyculatin S, acronyculatin T, mallotojaponin B, and lichenxanthone previously isolated from the *Mallotus oppositifolius* (Euphorbiaceae) [10] were tested for their activity against MRSA ATCC 33591. Only mallotojaponin B showed activity against MRSA; which was bactericidal according to Traoré and al. [21] (MBC/MIC ratio ≤ 4). In the perspective of linking activity to the structures of the compounds, it was noticed that all the four compounds do not really portray structural resemblance though mallotojaponin B could be regarded as a modified dimer of the acronyculatin S and T. Indeed, mallotojaponin B belongs to the large family of phenolic compounds which are known for their potential inhibition through the alteration of membrane structures or the inactivation of constituents or essential functions of the cell by different modes of action. Other modes of action involve the modification of membrane permeability, the modification of different intracellular functions induced by the hydrogen bonding of phenolic compounds to enzymes, or the modification of the cell wall which losses its integrity due to different interactions with the cell membrane [22, 23]. The bacterial susceptibility test was performed on several antibiotics (ampicillin, cloxacillin, oxacillin, gentamycin, chloramphenicol and penicillin) to which MRSA would have developed resistance phenomena according to literature review data [24–26]. The purpose of this test was to select the least effective antibiotic on MRSA ATCC 33591. Chloramphenicol being the least active with a MIC of 250 μg/mL, the corroborating previous result reported by Fankam *et al*. [25], was selected for combination and mode of action study.

Since combinatory therapy would be a promising alternative to effectively fight against the resistance phenomena [27], and mallotojaponin B was active against MRSA ATCC 33591 which itself is resistant to chloramphenicol, an evaluation of the activities of their combinations against MRSA was envisaged. At the end of this combination study, a synergistic effect was observed (FICI 0.393) with MIC reductions ranging from 12.5 μg/mL (MIC) to 0.781 μg/mL (1/16 MIC) for mallotojaponin B and from 250 μg/mL (MIC) to 1.95 μg/mL (1/128 MIC) for chloramphenicol. Given the knowledge of the mode of action of chloramphenicol, the mode of action of mallotojaponin B and the combination was investigated by evaluating the effect of these products on bacterial growth kinetics, salt tolerance, membrane permeabilization, and biofilm inhibition/eradication.

The resistance of most bacteria is due in part to the membrane which represents a semi-permeable barrier to the various antibiotics [28]. The measurement of the membrane permeability of bacteria is therefore essential in the study of the mode of action of antibiotics [29]. The literature reports that phenolic compounds have multiple cellular targets and can target different components and functions in the microbial cell, particularly the cytoplasmic membrane [30]. From our investigation, only combinations 2 and 3 in comparison with Triton X (positive control) resulted in an alteration of the membrane resulting in a significant release of nucleic acids into the extracellular medium. This could be explained by the ability of phenolic compounds to penetrate the phospholipid bilayer of the bacterial cell membrane to induce the modification of configuration either by depolarization, or by a chemosmotic perturbation, or an increase of the membrane permeability leading to a leakage of the cellular constituents including nucleic acids. The loss of salt tolerance observed in the presence of mallotojaponin B and synergistic combinations could be explained by the fact that these substances would alter osmotic phenomena leading to the entry of excess water into the cell with the consequence of cell rupture. Etame *et al*. [17] demonstrated that the loss of salt tolerance by MRSA after addition of a substance explains the ability of this substance to prevent the bacteria from expelling salts from the cells.

The formation of biofilms by *S*. *aureus* represents one of their best strategies for survival and resistance to antibiotics. It is one of the mechanisms of resistance that poses a real problem in the treatment of bacterial infections. Thus, the search for new antibacterial substances with the ability to destroy and/or inhibit biofilm formation would be of great benefit in the treatment of bacterial infections [31]. Biofilm inhibition and eradication tests were performed and the percentages of biofilm inhibition and eradication ranged from 31.75 to 75.13% and 13.56 to 64.50% respectively. It was found that the inhibition and destruction of biofilms are proportional to the concentration of the compound and combinations. The minimum concentrations that inhibits 50% of biofilm formation (MBIC_50_) and eradicates 50% of biofilm formed (MBEC_50_) for mallotojaponin B were 12.5 μg/mL and 50 μg/mL respectively. This activity was found to be comparable to that obtained with the synergistic combination (1.56 μg/mL mallotojaponin B; 3.9 μg/mL chloramphenicol) which inhibited and eradicated 73.97% and 50% of biofilms formed by MRSA respectively. These results corroborate those of Walencka *et al*. [32] who demonstrated that the combination of phenolic compounds (Salvipison and Aethiopinon) and antibiotics such as oxacillin act synergistically and inhibit the formation and eradication of MRSA biofilms probably by changing the hydrophobicity of the cell surface and the permeability of the cell wall/membrane. Also, the synergistic effect due to the combination of two phenolic compounds has been observed as is the case for salicylic acid and trans-cinnamaldehyde [33].

The search for new antibiotics for the treatment of pathologies due to multidrug-resistant bacteria is increasingly critical because of undesirable side effects such as cytotoxicity of isolated, semi-synthetic, or synthetic substances. However, antibacterial substances must have a higher benefit/risk ratio in favor of the benefits. For this purpose, the cytotoxicity evaluation is considered a preliminary test to ensure the safety of these substances. Thus, the safety of chloramphenicol and synergistic combinations was evaluated on cells of the Raw and Vero cell lines. At the end of this test, the percentages of inhibition of the latter varied from 8.065% to 30.328% and 0.00% to 19.50% respectively on Vero and Raw cells. According to the classification criterion defined by NCI, a substance is said to be non-cytotoxic if it presents a percentage of inhibition lower than 32% [20]. Therefore, just like chloramphenicol, no combination was cytotoxic on Vero and Raw cells.

## Conclusions

At the end of this study whose general objective was to evaluate the activity of the combination of Phloroglucinol with chloramphenicol and the mode of action of the synergetic combinations against methicillin-resistant *Staphylococcus aureus* ATCC 33591, it appears that: Methicillin-resistant *Staphylococcus aureus* ATCC 33591 is sensitive to mallotojaponin B (phloroglucinol) and resistant to chloramphenicol. The combination of mallotojaponin B and chloramphenicol has bactericidal synergistic effects on MRSA without toxic effects on Vero and Raw cell lines. mallotojaponin B and synergistic combinations act on MRSA by destabilizing and destroying the cell membrane and have the ability to inhibit and/or destroy biofilms formed by MRSA, making the combination a potential anti-staphylococcal drug candidate which would constitute a basis for the development of an alternative or complementary therapy against methicillin-resistant *Staphylococcus aureus*.
